# Death of a Child from Injury, Dependent on Forcible Exposure

**Published:** 1846-06

**Authors:** 


*Death of a Child from injury, Dependent on forcible Exposure.*—Mrs. B., aged 30, married, and pregnant with her first child, was seized during the night with labour pains. Being a refugee from the late fire in Quebec, she occupied part of a garret in which two or three other families, and some young men, were sleeping. Feeling a delicacy at being confined under such circumstances, she suppressed her cries till daylight, when she descended into a lower apartment, in which resided a woman who had been recently confined, to whom she detailed her feelings, requesting, at the same time, that some warm water might be given her to sit over, to relieve what she described as a great pressure at the lower part of the bowels. She had scarcely seated herself on the edge of a rather high chair, when a severe pain seized her, and before any assistance could be afforded, (though one or two women were in the room,) the child was forcibly expelled, and fell head foremost on the floor, being killed on the spot. The reporter of the case was sent for immediately after Mrs. B. had descended into the lower chamber, but did not arrive till about twenty minutes after the delivery. The child, which was a remarkably fine one, was perfectly dead, and still attached by the cord to the placenta, which came away shortly after the infant. In the above case not the slightest suspicion of criminality can attach to the mother; but suppose the delivery to have taken place in private, though there would be ground for a medico-legal investigation, still with the fact brought before them by the Coroner, that such cases as that now reported do not unfrequently occur, a jury should be extremely cautious how they return a verdict of wilful murder against the unfortunate mother.—*British American Journal.*

